# Effect of Temperature on the Mechanical Properties and Polymerization Kinetics of Polyamide-6 Composites

**DOI:** 10.3390/polym12051133

**Published:** 2020-05-15

**Authors:** Mei-Xian Li, Dasom Lee, Gyu Hee Lee, Seung Mo Kim, Goichi Ben, Woo Il Lee, Sung Woong Choi

**Affiliations:** 1 Center for Biomaterials, Korea Institute of Science and Technology, 5. Hwarang-ro 14-gil, Seongbuk-gu, Seoul 02792, Korea; lmx321@kist.re.kr; 2 Composites Research Division, Korea Institute of Materials Science (KIMS), 797, Changwon-daero, Seongsan-gu, Changwon-si, Gyeongsangnam-do 51508, Korea; dslee07@kims.re.kr; 3 School of Mechanical and Aerospace Engineering, Seoul National University, 1, Gwanak-ro, Gwanak-gu, Seoul 08826, Korea; gyuool@snu.ac.kr (G.H.L.); wilee@snu.ac.kr (W.I.L.); 4 School of Mechanical Engineering, Korea University of Technology and Education, Chungjeol-ro 1600, Byeongcheon-myeon, Dongnam-gu, Cheonan-si, Chungcheongnam-do, Cheonan 31253, Korea; smkim@koreatech.ac.kr; 5 Department of Mechanical Engineering, Nihon University, 2-chōme-1 Misakichō, Chiyoda City, Tōkyō-to 101-0061, Japan; ben.goichi@nihon-u.ac.jp; 6 Department of Mechanical System Engineering, Gyeongsang National University, 2, Tongyeonghaean-ro, Tongyeong-si, Gyeongsangnam-do 53064, Korea

**Keywords:** ε-caprolactam, vacuum assisted resin transfer molding (VARTM), differential scanning calorimeter (DSC), polymerization kinetics, crystallinity

## Abstract

This work reports the preparation of carbon fiber reinforced thermoplastic composites via the in situ anionic ring opening polymerization of ε-caprolactam. Vacuum assisted resin transfer molding was used to fabricate polyamide-6/carbon fiber composites at different molding temperatures. As a result, the higher polymerization of ε-caprolactam was observed with the condition at 140 °C for satisfactory impregnation. Regarding molding temperature, the physical properties of polyamide-6/carbon fiber were observed that the bending and impact strengths at 140 °C were higher than those to at other molding temperatures. The polymerization kinetics of polyamide-6 was analyzed using differential scanning calorimetry by experimentally acquiring kinetic parameters according to model fitting approaches. Polymerization and crystallization, which occur simultaneously throughout the whole process, were separated using Gaussian and Maxwell–Boltzmann distributions to study polymerization kinetics. The result of the developed model was in good agreement with the experimental data for the presented first order autocatalytic reaction model.

## 1. Introduction

Carbon fiber reinforced polymer (CFRP) composites have been widely used in industrial applications because of their high specific strength [1,2,3]. In particular, carbon fiber reinforced thermoplastic (CFRTP) composites are considered to be a better alternative for the automotive industry because of their potential for high processability and recyclability. However, with thermoplastic resin’s properties, such as high melt viscosity, have been considered unsuited for processing techniques requiring extensive resin flow, such as liquid composite molding processes (LCM). The problem with high resin viscosity may be solved if an in situ polymerization technique is employed. In this technique, monomers with catalysts and activators are polymerized during the resin injection process. Thermoplastic resin transfer molding (T-RTM) can also be incorporated with low viscosity of monomer, where the polymerization can successfully be applied to large structures. While rapid polymerization is regarded as having advantages leading to a reduction in cycle time, it is difficult to control the molding process because of the rapid polymerization time. There are several types of monomers that have low viscosities below 1 Pa·s, which are suitable for the liquid molding process. However, processing temperatures of the representative monomers such as polyamide-12 (PA-12), polyethyleneteraphthalate (PET), polybutyleneteraphthalate (PBT), and polycarbonate (PC) are too high (above 200 °C) for T-RTM. Although the processing temperature of polymethylenethacrylate (PMMA) is below 200 °C, its processing time is too long at low temperatures, while its monomer is easily boiled at high temperatures, resulting in voids in the composites. Therefore, the polyamide-6 (PA6) monomer, termed ε-caprolactam, is the most promising material among these thermoplastic precursors [4,5].

Research regarding the anionic polymerization of ε-caprolactam with various types and ratios of catalysts and activators has been studied [6,7,8,9,10,11]. Ahmadi et al. [6] investigated the reactive polymerization of ε-caprolactam inside an internal mixer using characteristic techniques with X-ray diffraction (XRD), gas chromatography (GC)-mass, differential scanning calorimetry (DSC), and thermal gravimetric analysis (TGA). For the PA6 sample, tensile strength and the impact properties were analyzed. Desai et al. [7] studied the synthesis process of nylon-6 from ε-caprolactam using an anionic polymerization method, through a two-step process using sodium metal as a catalyst and diisocyanate-caprolactam block initiator. Nagy et al. [8] investigated the characterization of the influence of both mold geometry and fiber orientation as influencing factors during injection and simultaneously occurring reaction on the production of fiber reinforced parts using anionic polymerization of ε-caprolactam. Barhoumi et al. [9] conducted a study of the kinetics of the anionic polymerization of ε-caprolactam into polyamide 6 through dynamic rheology and differential scanning calorimetry measurements to observe the effect of the processing parameters, such as the polymerization temperature and different catalyst/activator combinations and concentrations, on the kinetics of polymerization. Mohammadian-Gezaz et al. [10] examined activated anionic polymerization of caprolactam in the presence of styrene maleic anhydride (SMA). Here the effect of styrene maleic anhydride (SMA) on the anionic polymerization of ε-caprolactam was investigated. Kim et al. et al. [11] investigated the effect of poly(ethylene glycol) (PEG)-diamine on the overall rate of polymerization of caprolactam by fitting the experimental temperature rise with a new polymerization kinetic equation involving the polymerization exotherm, polymerization-induced crystallization exotherm, and the heat loss due to the adiabatic condition in the experimental situation. 

Meanwhile, the evaluation of mechanical properties for PA6 is a critical aspect in anionic polymerization. Therefore, extensive research has been conducted for the evaluation of material properties [12,13,14,15,16,17,18,19,20]. For instance, Meng et al. [12] investigated polyamide-6/graphite nanoflake (PA6/GnF) composites to obtain high thermal conductivity through a one-step in situ intercalation polymerization process. Zheng et al. [15] studied a one-step in situ polymerization and thermal reduction approach to prepare electrically conductive PA 6/reduced graphene oxide (RGO) nanocomposites evaluating stability of electric conductive and thermal conductivity. Tohidi et al. [20] examined the flexural and the impact properties of polyamide 6-based knitted reinforced single polymer composites (KSPCs) prepared by compression molding of powder-coated textile structures. Li and Shimizu [21] have evaluated properties using poly(vinylidene fluoride) (PVDA), polyamide 6 (PA6), and carbon nanotubes (CNTs) composites to examine the relationships between the physical properties (e.g., electrical conductivity and mechanical properties) and morphology of the composite. Li et al. [22] conducted a study of interfacial interaction, intercalation structure, and the synergistic reinforcing and friction-reducing effects of graphene nanosheets-3 aminopropyl-terminated poly(dimethylsiloxane) (GNs-APDMS) on MC PA6. Carey et al. [23] has dealt with a simple and scalable method of incorporating clay-like, multilayer Ti3C2Tz MXene into nylon-6, to study their barrier properties to permeating water vapor. Recently, Ben et al. manufactured glass fiber reinforced thermoplastic (GFRTP) composites and CFRTP composites with A (caprolactam and activator) and B (caprolactam and catalyst) mixtures and evaluated their mechanical properties [24,25]. 

However, few studies on polymerization kinetics and modeling related to experiments have been conducted compared to the wide range of studies of polymerization and evaluation of properties. Polymerization kinetics is critical in determining the reaction mechanism, therefore should be studied to control the molding processes and cycle time, especially for considering large scale products, since it mainly influences the final properties of the polymers.

In the present study, we focused on the effect of temperature on mechanical properties and polymerization kinetics for PA6. PA6 and CFRTP composites were manufactured at various molding temperatures via vacuum assisted resin transfer molding (VARTM). Moreover, polymerization kinetics was evaluated and modeled by separating the polymerization and crystallization stage from differential scanning calorimetry (DSC) data. 

## 2. Materials and Methods

### 2.1. Materials

**Figure 1:**
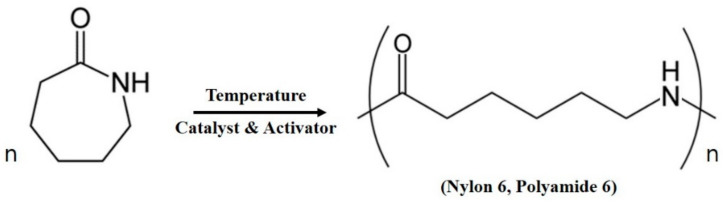
Anionic polymerization of ε-caprolactam.

The monomer, ε-caprolactam (DSM, Heerlen, Netherlands), which has a watery viscosity (3–4 mPa·s, 110 °C), was used in this study. It was mixed with a catalyst and an activator for anionic polymerization at specified molding temperatures, as shown in Figure 1. Sodium metal (DAEJUNG, Siheung-si, Korea) and hexamethylene diisocynate (HMDI) (Wako Chemicals, Wako-shi, Japan) were used as the catalyst and activator, respectively. The fiber used in this study was carbon fabric supplied by MUHAN COMPOSITES (Gwangju-si, Korea) with a 3K (3 thousands of filaments per tow) for tow specifications and a weight and thickness of 200 g/m^2^ and 0.22 mm, respectively.

### 2.2. Experimental Procedures

#### 2.2.1. Composite Specimen Prepared Using VARTM

**Figure 2:**
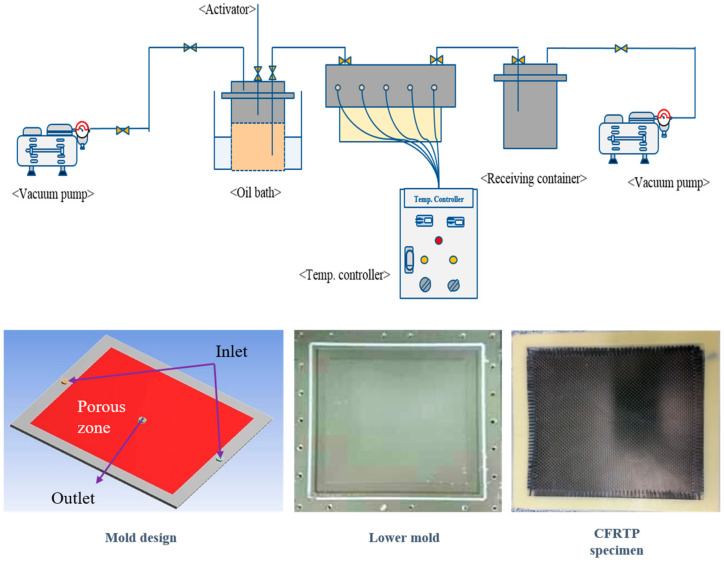
Schematic illustration of the VARTM process.

Manufacturing of the composite material was achieved with a VARTM process, and a schematic illustration of the experimental apparatus is shown in Figure 2. The monomer and catalyst were melted in an oil bath, and 13 layers of carbon fabric were piled into the mold before molding to maintain about 45% of fiber volume fractions. The monomer, ε-caprolactam, was dried at 40 °C for 24 h with silica gel blue in a vacuum oven. The catalyst, sodium metal for the anionic polymerization of ε-caprolactam, is sensitive to humidity in the air. Therefore, the container in the oil bath must remain in a vacuum condition during the melting process and the mold must be well-sealed. A pre-specified ratio of ε-caprolactam and sodium metal were melted at 110 °C for 1 h under a vacuum condition. As for the fiber, the carbon fabric was washed with acetone to remove the binding agent which may decrease the interfacial performance between the resin and fiber. It was dried at 80 °C for 5 min to completely remove the solvent prior to installation into the mold, according to Ben’s previous studies [24,25]. The dried carbon fabric was piled into the mold and the mold was sealed well before injection. After melting, the activator, HMDI, was injected into the mixture of the monomer and catalyst, and then transferred into the mold and heated for 5 min for polymerization. Because the polymerization temperature of thermoplastic resin is between 120 and 200 °C, the temperature condition of the mold was selected to be varied at 120 °C, 140 °C, 160 °C, 180 °C and 200 °C, respectively.

#### 2.2.2. Degree of Polymerization and Measurement of Water Absorption 

To evaluate the effect of different molding temperatures of in situ polymerized PA6 and CFRTP samples, the degree of polymerization was investigated with the content of the unreacted monomer and water absorption. Measurement of unreacted monomer of specimens at different molding temperatures was carried out to evaluate the degree of polymerization. Samples were manufactured at different molding temperatures with the size of 60 mm × 10 mm × 3.75 mm. Caprolactam (ε-caprolactam) monomer was placed in a three-neck flask and dried at 120 °C for 5 h under argon atmosphere in order to remove the moisture by less than 0.013 mol%. Each sample was dried at 60 °C for 24 h in a vacuum chamber, and its weight was measured and marked as *W*_0_. With dried samples, each was placed in hot water at 80 °C for 72 h, and the weight was recorded as *W*_2_. Finally, each sample was dried at 60 °C for 72 h in a vacuum chamber again, and its weight recorded as *W*_1_. With these data, we can obtain the content of the unreacted monomer and water absorption of the PA6 and CFRTP composites using Equations (1) and (2).

(1) $$W_{u}=\frac{W_{0}-W_{1}}{W_{0}}\times 100\%$$

(2) $$W_{a}=\frac{W_{2}-W_{1}}{W_{2}}\times 100\%$$

where *W_u_* is the content of the unreacted monomer, and *W_a_* is the content of water absorption.

#### 2.2.3. Three Point Bending and Impact Test 

Three point bending test specimens (100 mm × 13 mm × 3.75 mm) were fabricated with the 60 mm of span length according to American society for testing and materials (ASTM) D790-10 specifications, to measure the bending properties of flexural strength and flexural modulus of the pure PA6 and CFRTP composite samples fabricated with different molding temperatures. The three point bending test was conducted with an Instron-5584 (Instron, Buckinghamshire, UK) universal materials testing machine. The experiment was conducted at least nine times under each condition, and the results are shown with an error bar. Impact tests were conducted to evaluate the impact strength of the specimen using an Izod pendulum-type impact apparatus (SALT, Incheon, Korea) with a maximum capacity of 60 kgf·cm. Test samples were fabricated in accordance with ASTM D256 [26], which is a standard method of testing Izod pendulum impact resistance of plastics to obtain the impact strength of the pure PA6 and CFRTP composite samples fabricated with different molding temperatures. Each specimen had dimensions of 61.5 mm height, 11.5 mm depth, 3.75 mm width, and 2.54 mm notch depth. The experiment was conducted at least eleven times under each condition, and the results are shown with an error bar. 

#### 2.2.4. SEM Observation

Fractured surface of the CFRTP specimen was observed with SEM (FE-SEM, 7800F Prime, JEOL Ltd., Tokyo, Japan). The fractured specimen for the observation was 50 mm × 13 mm × 3.75 mm obtained from the impact test piece to evaluate the interfacial adhesion between the fibers and the resin. The sample was coated with gold before scanning to avoid surface charge, and the accelerating voltage was lower than 20 kV to prevent sample degradation. 

#### 2.2.5. DSC Measurement

The polymerization behavior and kinetics of ε-caprolactam was evaluated and modeled using DSC (DSC-Q 1000, TA Instrument, Hertfordshire, UK) to observe the relationship between the temperature and polymerization for both the dynamic and isothermal scanning processes. DSC measurement was conducted with the sample prepared as follows: First, ε-caprolactam was dried at 40 °C for 24 h with silica gel blue in a vacuum oven to remove moisture. Subsequently, the dried ε-caprolactam and sodium metal were melted at 110 °C in the oil bath for 1 h under a vacuum condition. Thereafter, the ε-caprolactam and sodium metal mixture were cooled to 70 °C following the addition of the activator, and the mixture was transferred to a glass vial and sealed. This sealed vial was rapidly cooled in liquid nitrogen to prevent polymerization before the DSC scans. Samples weighing approximately 10 mg were sealed in aluminum pans for both the dynamic and isothermal scanning processes.

## 3. Results and Discussion

### 3.1. Measurement of Content of Unreacted Monomer and Water Absorption 

**Figure 3:**
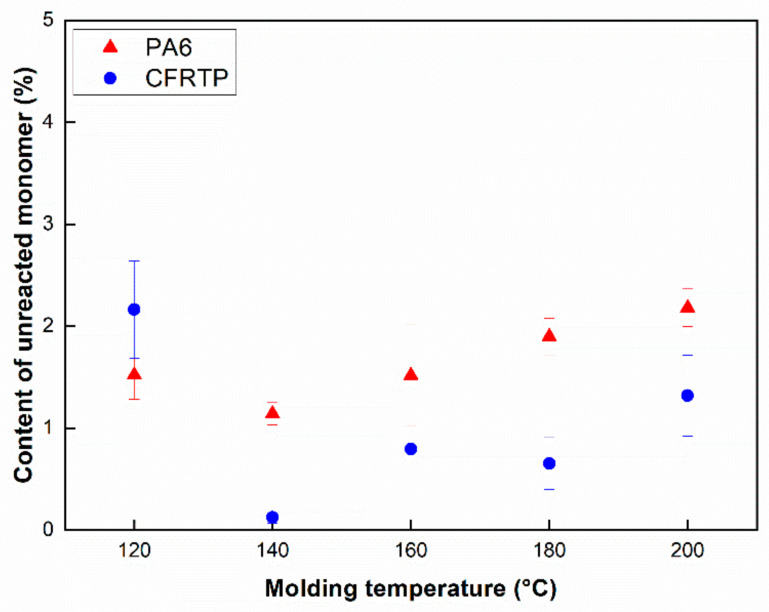
Unreacted monomer content of the pure PA6 and CFRTP composites.

**Figure 4:**
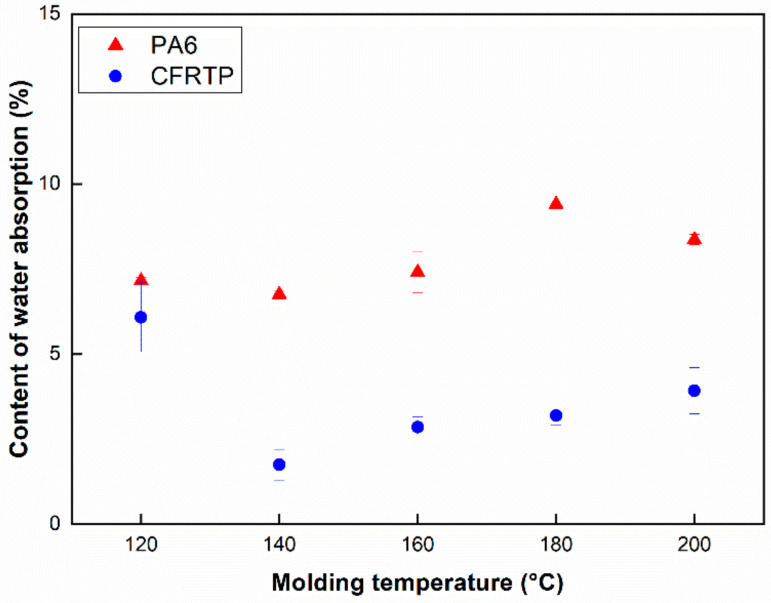
Water absorption content of the pure PA6 and CFRTP composites.

The relationship between the content of the unreacted monomer and the water absorption of pure PA6 and CFRTP composite is investigated to observe the conversion of ε-caprolactam, demonstrating its polymerization process, shown in Figure 3 and Figure 4. In Figure 3, the percentage of the unreacted monomer (*W_u_*) of the pure PA6 and CFRTP composite is shown as a function of the molding temperature. It can be seen that the content of the unreacted monomer for both the pure PA6 and the CFRTP composites shows its lowest value at 140 °C. Figure 4 shows the content of the water absorption (*W_a_*) of the pure PA6 and CFRTP composites. It can be also seen that the content of the water absorption (*W_a_*) for both the pure PA6 and the CFRTP composites shows its lowest value at 140 °C, which showed the same tendency as the content of unreacted monomer. Both the pure PA6 and CFRTP composites show their lowest content of water absorption value at 140 °C. In addition, the contents of both the unreacted monomer and water absorption increased with increasing molding temperature. For ε-caprolactam it can be said that polymerization and crystallization occurred simultaneously. At 120 °C, it was shown that crystallization is dominant. Trapped unreacted monomers due to the crystallization can interfere with the polymerization process, resulting in more unreacted monomers. Therefore, it can be shown that there exists an optimal temperature region for the polymerization of ε-caprolactam, which may be between 140 °C and 160 °C from these results [27]. Furthermore, the contents of the unreacted monomer and the water absorption of the pure PA6 and the CFRTP exhibit a similar behavior with molding temperatures, which may indicate that the carbon fabrics which were used as reinforcement rarely affect the polymerization of ε-caprolactam. This is because a certain amount of unreacted monomer still exists, which is very hydroscopic and is readily dissolved in water after polymerization. Relatively large deviations in Figure 3 and Figure 4 can be attributed to the amount of polymerization and crystallization. A relatively small amount of polymerization and crystallization can affect the amount of unreacted monomer and the amorphous part, indicating large deviations of properties.

### 3.2. Three Point Bending and Impact Test

**Figure 5:**
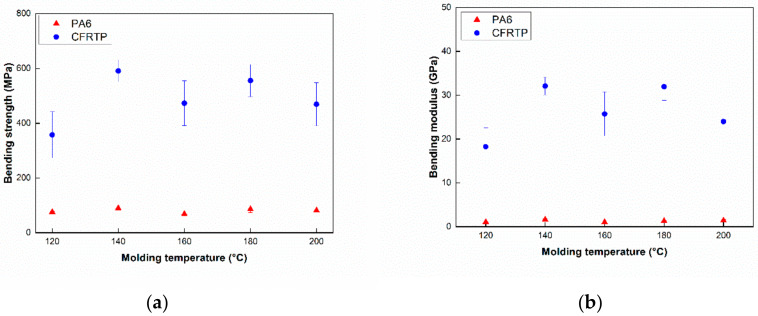
Bending strength (**a**) and modulus (**b**) of the pure PA6 and CFRTP composites at different molding temperatures.

Due to the importance of the bending properties of CFRTP composites for automotive products, bending strength and bending modulus was obtained with a three point bending test. Figure 5 shows the change of the bending test results of the pure PA6 and the CFRTP composites with respect to the molding temperatures. The values of bending strength and bending modulus are shown for the CFRTP composites (350–600 MPa) and PA6 (60–90 MPa) and CFRTP composites (15–35 GPa) and PA6 (1–2 GPa), respectively, showing relatively large variations of CFRTP composites compared to the PA6. The values of bending strength and modulus was found to be highest at 140 °C for the pure PA6 and the CFRTP composites. These results could be explained by reacted monomer and degree of water absorption of the composite. The composite with highest content of the reacted monomer and a low degree of water absorption was shown to have higher values of bending strength and modulus. For the molding temperature of 140 °C, the higher values of bending strength and modulus were measured, which was also identified with the previous findings [24].

**Figure 6:**
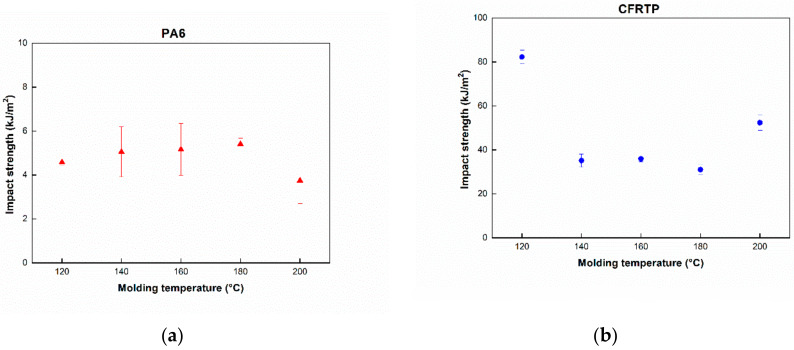
Impact strength at different molding temperatures. (**a**) PA6, (**b**) CFRTP.

The Izod impact tests for the pure PA6 and CFRTP composites were performed to evaluate the impact properties with respect to molding temperatures. Impact test results are shown in Figure 6, indicating that the impact strength of PA6 and CFRTP composites can be estimated to be approximately 3–6 kJ/m^2^ and 30–85 kJ/m^2^. The impact strength of the pure PA6 is shown to be highest at a molding temperature of 160 °C, whereas that of pure PA6 at molding temperatures of 120 °C and 200 °C is lower than that of other samples. This phenomenon could be due to the unreacted monomer (Figure 6a). However, for the impact strengths of the CFRTP composites (Figure 6b), they showed much higher values than those of others at molding temperatures of 120 °C and 200 °C. It may be that the impregnation of the carbon fibers at 120 °C and 200 °C is poorer than that of the others, and there is much unreacted monomer when it is molded at 120 °C and 200 °C. Specimens of CFRTP at 120 °C and 200 °C showed a partial break mode, while the others showed a complete break mode. These results further confirm why the impact strength of CFRTP at 120 °C and 200 °C shows higher values than specimens at other temperatures. CFRTP at 140–180 °C showed a complete break mode, resulting in relatively low impact strength. Large deviations in Figure 5 and Figure 6 results could also be attributed to the amount of polymerization and crystallization: these tendencies were identical with the results in Figure 3 and Figure 4.

### 3.3. Scanning Electron Microscopy Observation

**Figure 7:**
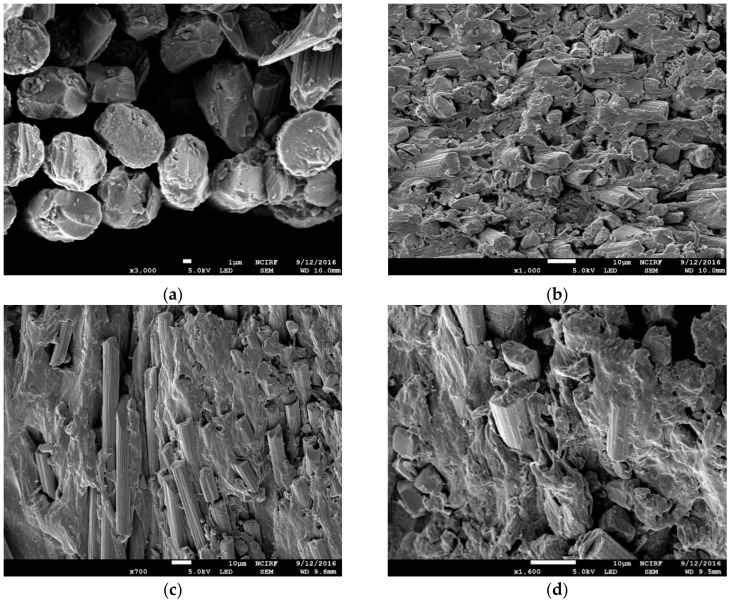

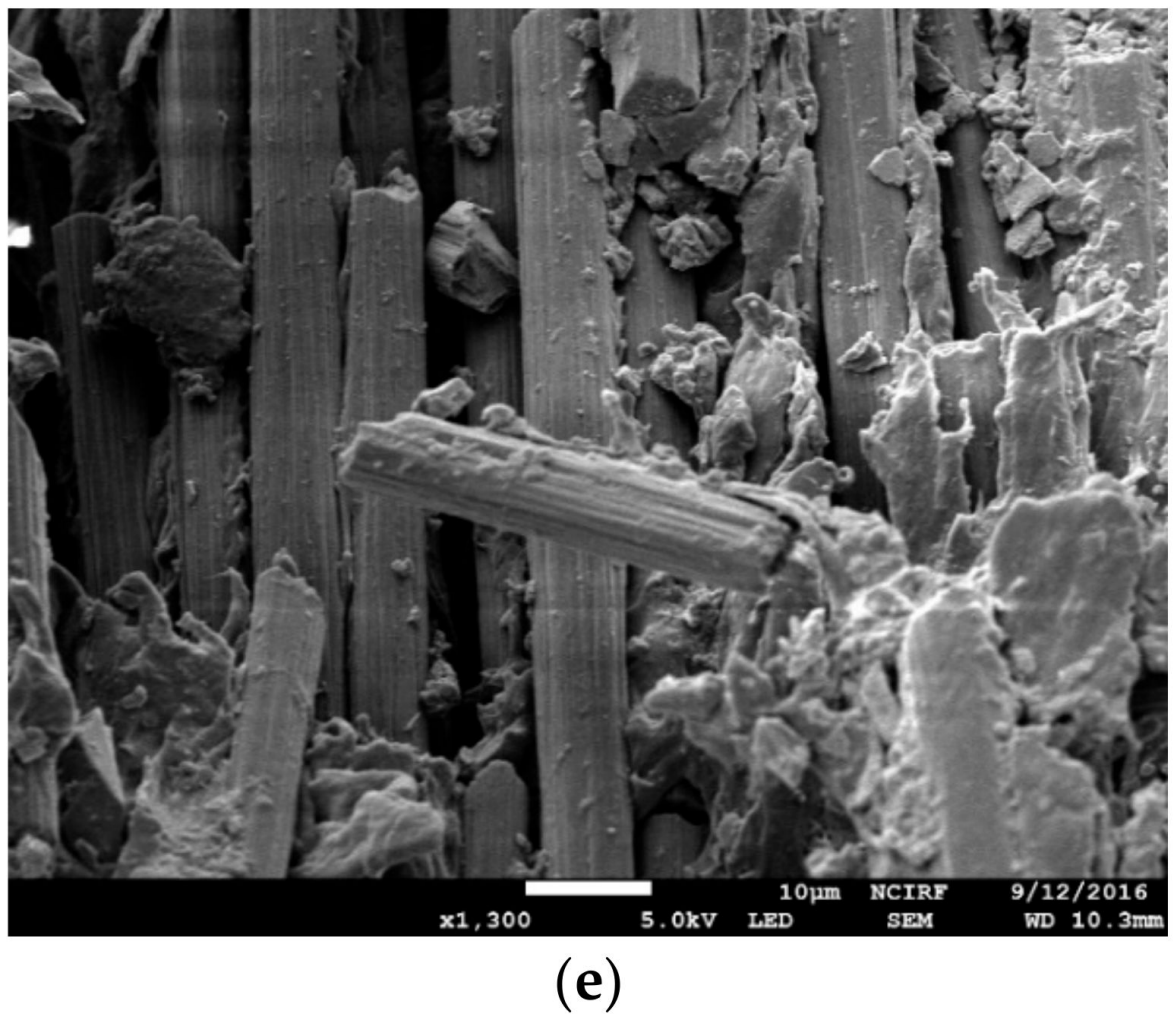
SEM micrograph of the fracture surface of the CFRTP composite at different molding temperatures: (**a**) 120 °C, (**b**) 140 °C, (**c**) 160 °C, (**d**) 180 °C, and (**e**) 200 °C.

To evaluate the adhesion between the fibers and the resin, the fracture surfaces of the CFRTP specimens molded at different molding temperatures were observed with scanning electron microscopy (SEM). Figure 7a–e show that the resin is homogenously impregnated around the carbon fabrics which were molded at 140 °C, 160 °C, and 180 °C. However, there is much resin around the carbon fabrics which were molded at 120 °C and 200 °C because of unreacted monomer. The results correlate with the findings on the mechanical properties of the specimens in each case.

From the results for all the mechanical properties tested herein, we can conclude that the optimal molding temperature for polymerization is between 140 °C and 160 °C.

Investigation of polymerization kinetics: the degree of polymerization and crystallinity are the main factors that greatly affect the mechanical properties of the samples. During the process, polymerization and crystallization take place simultaneously, and thus the sum of both contributions is reflected in the DSC data. In order to model the polymerization kinetics, the contribution from polymerization should be separated from the DSC data.

#### 3.3.1. Dynamic Analysis

**Figure 8:**
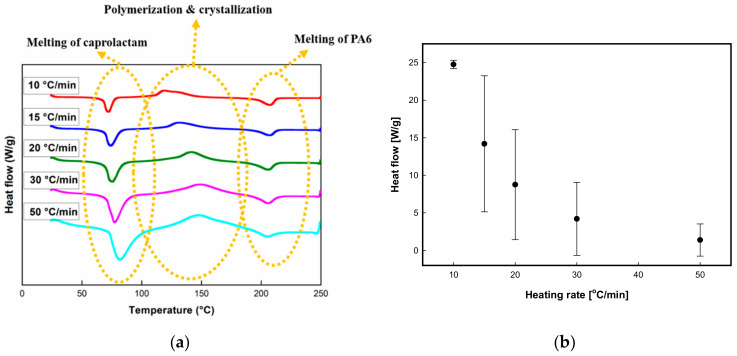
DSC thermograms of caprolactam (**a**): DSC thermograms of caprolactam at five different heating rates, (**b**): normalizing heat flow at different heating rates).

DSC samples were heated from room temperature to 250 °C at a heating rate of 10, 15, 20, 30, and 50 °C/min, respectively. Figure 8 showed the DSC thermograms of caprolactam with different heating rates and heat flow was normalized at different heating rates. As shown in Figure 8, the first peak corresponds to the melting of ε-caprolactam at approximately 70 °C, and the second peak shows the polymerization and crystallization peak during the heating process. During this process, polymerization and crystallization occur simultaneously, and both are exothermic processes [28,29,30]. The third peak represents the melting of PA6, which is related to crystallization during the heating process [31].

**Figure 9:**
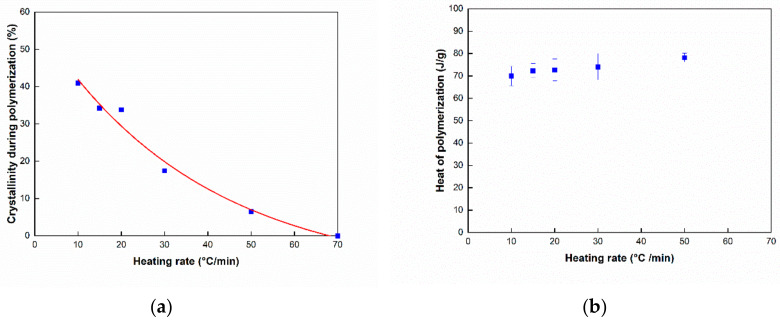
(**a**) Crystallinity at different heating rates during polymerization, (**b**) polymerization at different heating rates.

Using the second and third peaks, the heat of fusion of crystallization and polymerization can be estimated, and these results are shown in Table 1. Figure 9 shows crystallinity during polymerization and heat of fusion of polymerization with different heating rates. As can be seen in Figure 9a, the crystallization occurring simultaneously with polymerization decreased upon increasing the heating rate. This is probably because the synthesized polymer was not provided sufficient time to crystallize, and consequently, it reached its melting point before completing crystallization. Furthermore, the heat of fusion for polymerization can also be calculated as shown in Figure 9b. 

**Separation of polymerization and crystallization peaks:** Because the polymerization and crystallization of ε-caprolactam occur simultaneously, the exothermic peak shown in Figure 9 can be decomposed into the crystallization and the polymerization peaks. In this study, it was assumed that the polymerization peak follows the Gaussian distribution (Equation (3)), whereas the crystallization peak follows the Maxwell–Boltzmann distribution (Equation (4)).

(3) $$f\left(x\right)=\frac{1}{\sigma \sqrt{2\pi }}e^{-\frac{{\left(x-\mu \right)}^{2}}{2\sigma^{2}}}$$

where *µ* is the mean or expectation of the distribution, *σ* is the standard deviation, and *σ^2^* is the variance.

(4) $$f\left(v\right)=f\left(v\right)\sqrt{{\left(\frac{m}{2\pi kT}\right)}^{3}}\times \pi v^{2}e^{-\frac{mv^{2}}{2kT}}$$

where *m* is the particle mass, *k* is Boltzmann’s constant, and *T* is the thermodynamic temperature. 

**Figure 10:**
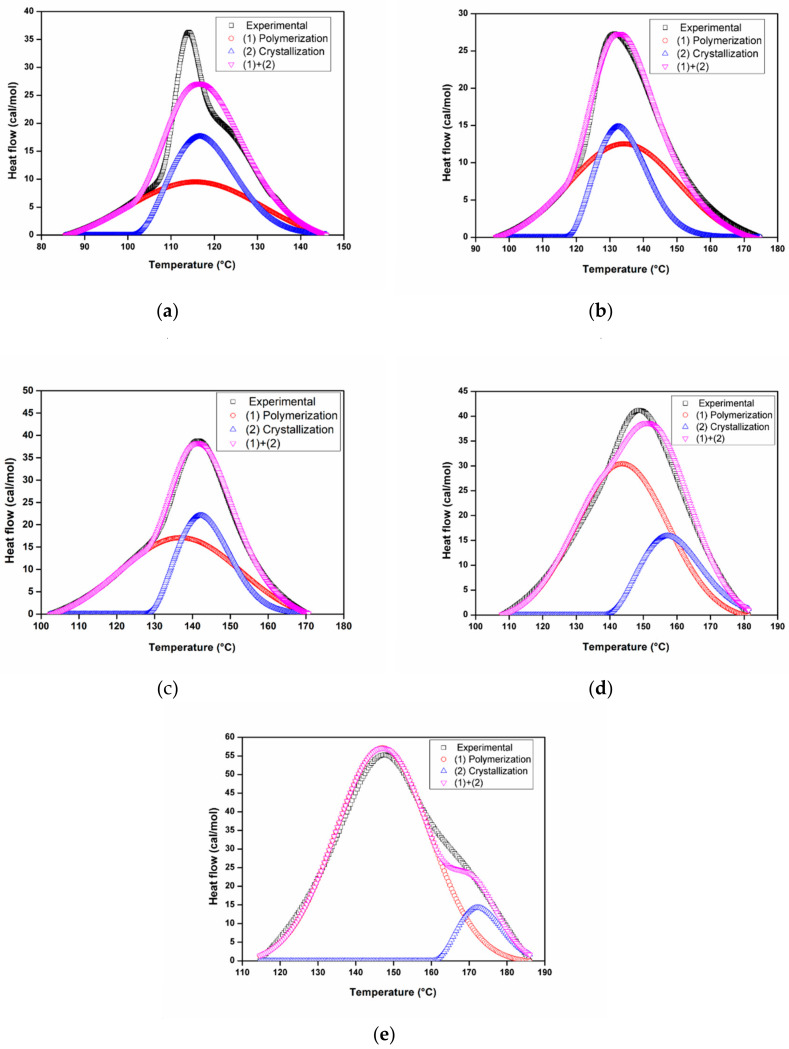
Separation of the polymerization and crystallization curves at different heating rates: (**a**) 10 °C/min, (**b**) 15 °C/min, (**c**) 20 °C/min, (**d**) 30 °C/min, (**e**) 50 °C/min.

Figure 10 shows the results from the separation of polymerization and crystallization peaks using the Gaussian and the Maxwell-Boltzmann distributions. As can be seen, the fitting data were in good agreement with the experimental results at different heating rates.

**Modeling of polymerization kinetics at different heating rates:** The non-isothermal kinetics of caprolactam activated anionic polymerization in bulk is known to be described by an Arrhenius type autocatalytic model [19,20]:

(5) $$\frac{d\alpha }{dt}=A_{0}\exp\left(-\frac{E}{RT}\right){\left(1-\alpha \right)}^{n}\;\left(1+B_{0}\alpha \right)$$

where *A_0_* is the pre-exponential factor, *E* is the activation energy, *n* is the order of the reaction order, *B_0_* is the autocatalytic factor, and *α* is the degree of conversion.

We chose the simplest form of the modeling equation that can fit the actual experimental data well:

(6) $$\frac{d\alpha }{dt}=A_{0}\exp\left(-\frac{E}{RT}\right)\left(1-\alpha \right)\;\left(1+B_{0}\alpha \right).$$

In the above equation, there are three parameters, *A_0_*, *E*, and *B_0_*. The values of these parameters were found by fitting the model equation to the data (Table 2).

**Figure 11:**
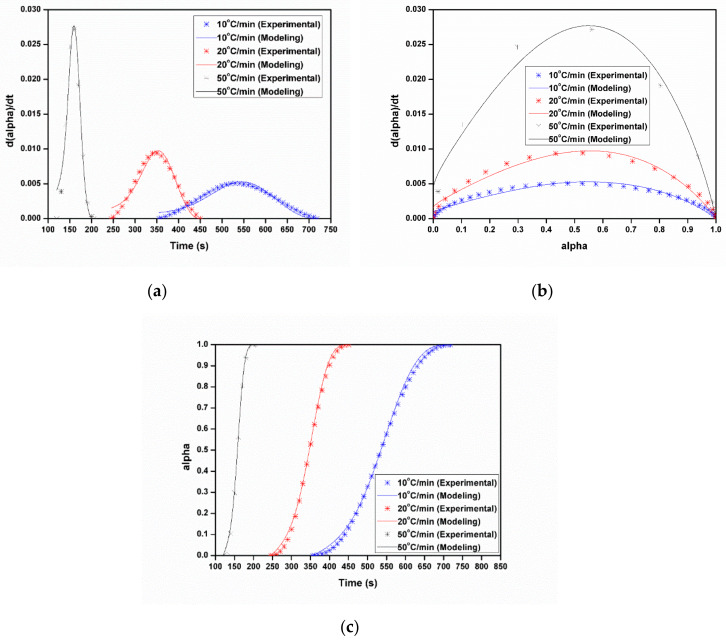
Comparison of modeling results to experimental data using the first order autocatalytic reaction model: (**a**) 10 °C/min, (**b**) 20 °C/min, (**c**) 50 °C/min.

Figure 11 shows the comparison between experimental and the first order autocatalytic reaction model for the relationship among the polymerization rate (*dα/dt*), time (*t*), and the degree of conversion (*α*) at different heating rates. The fitting curves match very well the experimental data; in other words, the first order autocatalytic reaction model seems suitable for the non-isothermal polymerization of ε-caprolactam.

**Figure 12:**
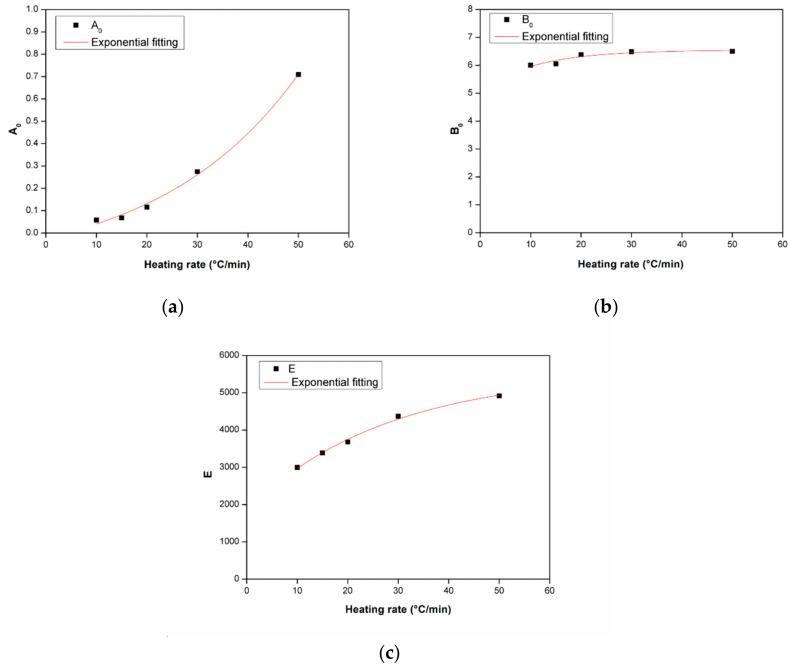
Kinetic parameters A_0_ (**a**), B_0_ (**b**), and E (**c**) for polymerization of ε-caprolactam at different heating rates.

**Figure 13:**
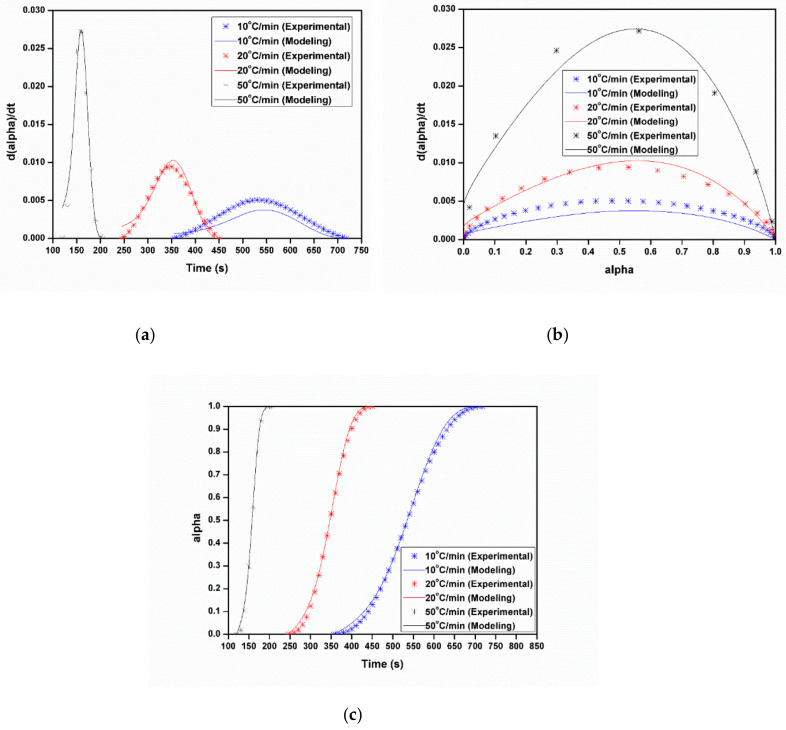
Comparison of modeling results to experimental data using exponential fitting parameters: (**a**) 10 °C/min, (**b**) 20 °C/min, (**c**) 50 °C/min.

Figure 12 shows the kinetic parameters for polymerization of ε-caprolactam at different heating rates, and these results are shown in Table 3. A comparison between experimental and modeling results using exponential fitting parameters is shown in Figure 13, indicating the modeling results matched with the experimental data closely. It can be suggested that the first order autocatalytic reaction model is suitable for describing the non-isothermal polymerization of ε-caprolactam.

#### 3.3.2. Isothermal Analysis

To investigate the polymerization kinetics of ε-caprolactam at different molding temperatures, a series of measurements of isothermal scanning were performed. First, DSC samples were kept at a specified molding temperature for 5 min and then cooled down to room temperature. Thereafter, samples were heated from room temperature to 250 °C at a heating rate of 10 °C/min to evaluate the degree of polymerization and the crystallinity during isothermal scanning. 

**Figure 14:**
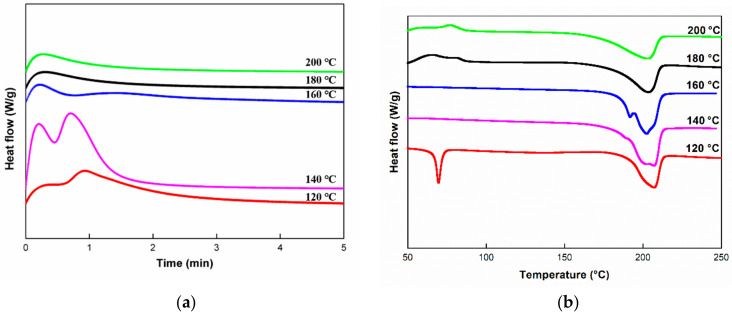
DSC thermograms of caprolactam with different polymerization temperatures: (**a**) isothermal scanning as a function of polymerization time, (**b**) dynamic scanning as a function of temperature.

Figure 14a,b shows the isothermal DSC thermograms of caprolactam as a function of polymerization time and dynamic DSC thermograms as a function of temperature with a molding temperature of 120, 140, 160, 180, and 200 °C, respectively. At lower temperatures (120–140 °C), crystallization and polymerization occur simultaneously, because fast crystallization results in the monomer being trapped inside crystals before polymerization [9]. However, at higher temperatures, the growth of chains is more significant than the formation of crystals, and thus, the molding temperature adversely affects both polymerization and crystallization rates. Therefore, there exists an optimal molding temperature for the best mechanical properties. 

The isothermal heat of reaction obtained by integrating the DSC flow curves with each reaction temperature is shown in Table 4. As shown in Table 4, the heat of reaction for isothermal scanning increases at lower temperatures (120–140 °C) and then decreases as the molding temperature increases. Referencing the dynamic scanning result (Figure 14b) shows that much unreacted monomer exists after isothermal scanning for 5 min at 120 °C, whereas no endothermic peak appears at other temperatures. In other words, most monomers were converted to PA6 at all the molding temperatures except for 120 °C. Furthermore, at lower molding temperatures (140–160 °C), a small exothermic peak occurs at approximately 70 °C because of full crystallization during the isothermal process. However, samples which were isothermally heated at high temperatures (180–200 °C) were recrystallized during the heating process, because the polymerization rate was higher than the crystallization rate at high temperature; therefore, few crystals can be formed at high temperature. 

**Determination of process window:** It can be identified that the polymer melting temperature slightly decreased with an increase in molding temperature (Table 5), implying that the crystals formed during isothermal scanning led to a higher melting temperature. Because ε-caprolactam is polymerized in a short time, the overall process must be completed within a critical processing time, as the viscosity of polymerizing ε-caprolactam increases rapidly. Since the process window is of utmost importance, especially when molding large parts, in this study, a critical processing window was defined. Generally, the fiber reinforcements in the mold for resin transfer molding can be regarded as a porous media. 

**Figure 15:**
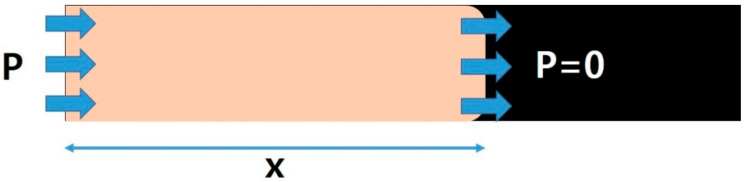
Illustration of porous media flow.

When the resin is injected into a mold, it flows via small gaps between the fibers. The most generally accepted equation to describe the flow in porous media is Darcy’s law (Equation (7)). Fluid flow for porous media can be described in a simple form with Equation (7) as follows:

(7) $$V=-\frac{K}{\mu }\frac{dP}{dx}$$

where *V* is velocity of the flow, *K* is the permeability of the medium, *µ* is the viscosity of the polymerizing ε-caprolactam, which is dependent on time and temperature, *P* is the pressure, and *x* is the displacement in the flow direction (Figure 15). The velocity of the flow *V* is equivalent to the advancing speed of flow front.

(8) $$V=\frac{dx_{f}}{dt}$$

where *x_f_* is the location of the flow front. 

From Equations (7) and (8), we have

(9) $$\frac{dx_{f}}{dt}=-\frac{K}{\mu }\frac{dP}{dx_{f}}$$

The relationship between the viscosity and the location of the flow front can be obtained by integrating the above equation with respect to time until the resin gels.

(10) $$\int_{0}^{t_{gel}}\frac{1}{\mu }dt\;\propto \;x_{f}{}^{2}$$

Once we have the viscosity behavior as a function of time during the process, we can estimate the maximum size of the molded part. Dave et al. [32] reported that below 50% polymerization conversion, the complex relative viscosity ($\left|\eta^{*}\right|$/$\left|\eta_{0}^{*}\right|$) values were almost linear in relation to the polymerization conversion, and the viscosity of ε-caprolactam monomer can be described by Equations (11) and (12).

(11) $$\left|\eta^{*}\right|=\left|\eta_{0}^{*}\right|\exp\left(kX\right)\;\left(X<0.5\right)$$

where $\left|\eta^{*}\right|$ is the complex viscosity of PA6 anionically polymerizing in its monomer, $\left|\eta_{0}^{*}\right|$ is the complex viscosity of the caprolactam monomer, and *k* is a constant. The change in the viscosity of the monomer during polymerization is dependent on the polymer fractional conversion *X*, where *X* can be obtained as a function of time from Figure 14. Fractional conversion *X* is attributed to the formation and growth of polymer chains. The complex viscosity of the monomer, $\left|\eta_{0}^{*}\right|$, follows an Arrhenius temperature dependence as follows:

(12) $$\left|\eta_{0}^{*}\right|\left(T\right)=2.7\times 10^{-7}\exp\left(3525/T\right)\left(\mathrm{Pa}\cdot s\right)$$

**Figure 16:**
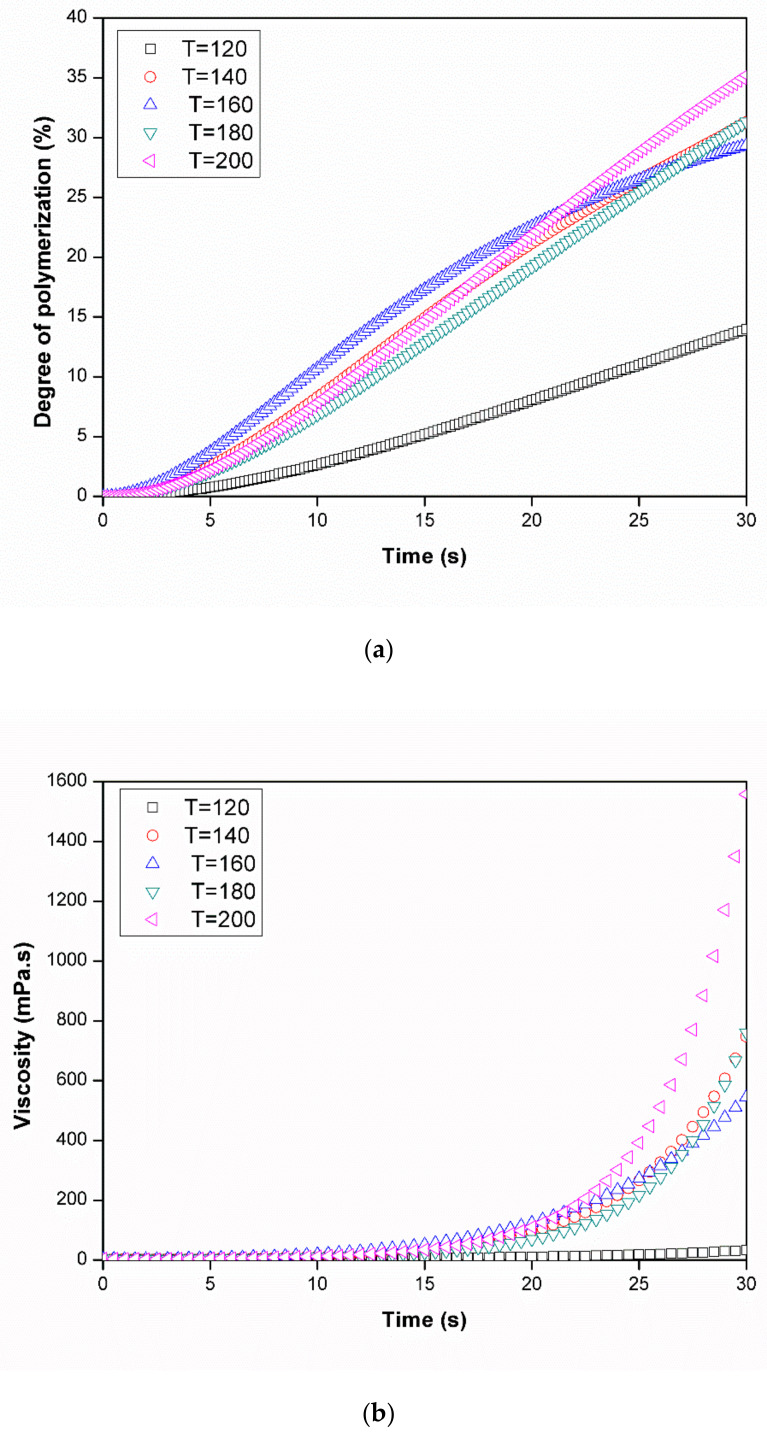
Degree of polymerization profile (**a**) and viscosity profile (**b**) of ε-caprolactam during the first 30 s at different temperatures.

Figure 16 shows the time dependent viscosity of the polymerizing ε-caprolactam at specific temperatures, calculated based on Equations (11) and (12) and the results of Figure 14. During the early stage, viscosity varies almost linearly with time; however, at 200 °C, it rapidly increases from 20 s onward. As can be expected, the viscosity at 120 °C increases slowly, as the polymerization rate is lower than that of the other cases.

**Figure 17:**
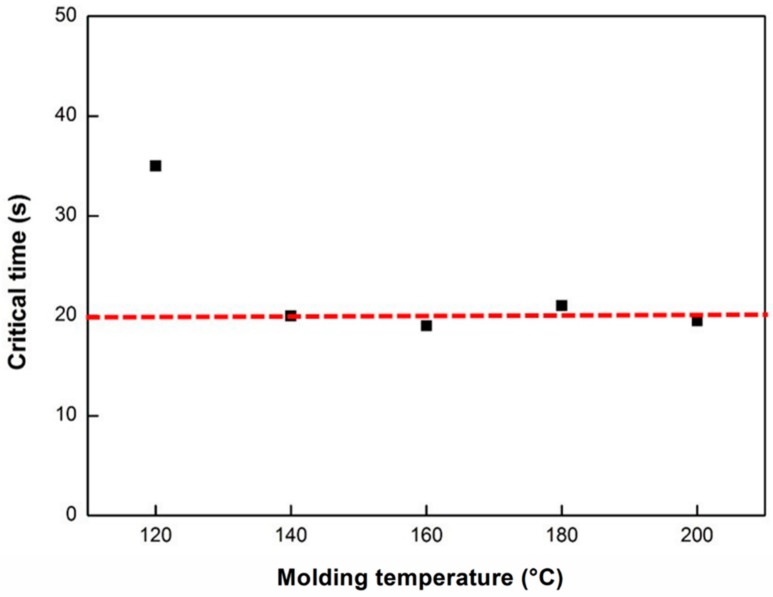
Critical time at different molding temperatures.

As mentioned above, the viscosity of polymerizing ε-caprolactam changes with time. Thus, we can obtain the relationship between the filling time and Equation (10), from which we can determine the critical time at different molding temperatures (Figure 17). The time when the location of the flow front becomes standstill can be defined as the critical processing time, and it can be shown that the molding process must be finished within this critical processing time (20 s) regardless of the shape and size of the parts.

## 4. Concluding Remarks

In this study, CFRTP via the in situ anionic ring opening polymerization of ε-caprolactam was prepared to investigate the effect of molding temperatures. PA6 and CFRTP composites were manufactured at various molding temperatures via vacuum assisted resin transfer molding (VARTM). For the effect of molding temperature, mechanical properties and polymerization kinetics for the PA6 was examined. Polymerization kinetics was evaluated and modeled by separating the polymerization and crystallization stage from DSC observations. The main conclusions of the study are as follows.

The content of unreacted monomer and the water absorption of CFRTP composites were at their lowest values when the samples were molded at 140 °C; at this temperature, the samples also exhibited the best bending strength and modulus. It is noted, however, that the impact strengths at 120 °C and 200 °C were higher than those at other temperatures, because of the high content of unreacted monomer and poor impregnation. 

The mechanical properties with respect to the molding temperature can be identified with the fractured surface of the CFRTP specimen using SEM observation. Homogenously impregnated resin around the carbon fabrics was found molded at 140 °C, 160 °C, and 180 °C, and resin spread around the carbon fabrics molded at 120 °C and 200 °C due to unreacted monomer.

In dynamic analysis, DSC thermograms of caprolactam with different heating rates was observed with peaks showing that polymerization and crystallization occur simultaneously, and crystallinity during polymerization decreases with increasing heating rate because of the insufficient crystallization time. 

According to the isothermal scanning results, crystallization and polymerization occurs simultaneously at lower temperature because at lower reaction temperatures monomer can be trapped inside crystals before being able to polymerize. However, at higher temperatures, the growth of the chain is more significant than the crystal formation. Therefore, the polymerization rate is higher than the crystallization rate at higher temperatures, and as a result, crystallinity is lower at higher temperatures.

To investigate polymerization kinetics, the polymerization peak was separated from the exothermic peak using Gaussian distribution. We used the first order autocatalytic reaction model to model the polymerization kinetics. This resulted in a very good fit to the data, and thus the model is found to be suitable for describing the polymerization kinetics of ε-caprolactam. 

From the experimental and modeling results, it can be concluded that the optimal temperature for polymerization is 140–160 °C, and the molding process must be finished within 20 s regardless of the shapes and sizes of the samples.

The present research regarding PA6 composites with molding temperature effect could be effectively used in future research regarding a wide range of design processes and numerical approaches for the manufacturing method. Our future research will be focused on PA6 dispersion with nanoparticles to improve impact and tensile properties, as well as radiant heat.

## Figures and Tables

**Table 1:** Values of released heat during the heating process.

Heating Rate (°C/min)	Melting of Caprolactam	Polymerization & Crystallization	Melting of PA6		Polymerization
	Released Heat (J/g)	Released Heat (J/g)	Released Heat (J/g)	Crystallinity (%)	Released Heat (J/g)
**10**	113.4	147.6	77.65	41.00	69.95
**15**	114.3	137.2	64.81	34.25	72.35
**20**	119.5	136.6	63.92	33.79	72.68
**30**	119.7	106.2	32.12	17.44	74.10
**50**	124.3	90.34	12.15	6.480	78.19

**Table 2:** Reaction parameters of polymerization kinetics at different heating rates.

Heating Rate (°C/min)	*A*_0_	*E*	*B*_0_
**10**	0.058	3000	6.005
**15**	0.068	3380	6.054
**20**	0.116	3680	6.382
**30**	0.274	4370	6.488
**50**	0.709	4920	6.498

**Table 3:** Reaction parameters of polymerization kinetics at different heating rates using exponential curves of Figure 12.

Heating Rate (°C/min)	*A*_0_	*E*	*B*_0_
**10**	0.041	2970	5.963
**15**	0.082	3390	6.175
**20**	0.131	3750	6.310
**30**	0.261	4290	6.450
**50**	0.711	4940	6.530

**Table 4:** DSC diagrams of the isothermal scanning process.

Molding Temperature (°C)	120 °C	140 °C	160 °C	180 °C	200 °C
Heat of reaction (J/g)	94.48	144.0	94.29	37.80	36.18

**Table 5:** DSC diagrams of the heating process.

Molding Temperature (°C)	Endothermic Reaction (J/g)	Melting (J/g)	*T*_m_ (°C)
120	−16.36	51.57	206.9
140	1.120	81.69	206.7
160	0.430	76.33	202.4
180	24.92	61.54	203.1
200	23.02	62.23	202.3
